# Supplementary material for: A GABAergic and peptidergic sleep neuron as a locomotion stop neuron with compartmentalized Ca2+ dynamics
Source: Nat Commun. 2019 Sep 10;10:4095. doi: 10.1038/s41467-019-12098-5 (PMC6736843; doi:10.1038/s41467-019-12098-5)
Supplement: Supplementary file 1 — Supplementary Information [file 41467_2019_12098_MOESM1_ESM.pdf]

# A GABAergic/peptidergic sleep neuron also functions as a locomotion stop neuron with compartmentalized $Ca^{2+}$ dynamics

Wagner Steuer Costa[1,2,10], Petrus Van der Auwera[1,2,5,10], Caspar Glock[1,2,6], Jana F. Liewald[1,2], Maximilian Bach[1,2], Christina Schüler[1,2], Sebastian Wabnig[1,2,7], Alexandra Oranth[1,2], Florentin Masurat[3], Henrik Bringmann[3,4], Liliane Schoofs[5], Ernst H. K. Stelzer[1,8], Sabine C. Fischer[1,8,9], Alexander Gottschalk[1,2,11]

1 Buchmann Institute for Molecular Life Sciences (BMLS), Goethe University, Max-von-Laue-Strasse 15, D-60438 Frankfurt, Germany

2 Institute for Biophysical Chemistry, Goethe University, Max-von-Laue-Strasse 9, D-60438 Frankfurt, Germany

3 Max Planck Institute for Biophysical Chemistry, Am Fassberg 11, 37077 Göttingen, Germany

4 Department of Biology, University of Marburg, Karl-von-Frisch-Strasse 8, D-35043 Marburg, Germany

5 Functional Genomics and Proteomics Group, Department of Biology, KU Leuven, Naamsestraat 59 - box 2465, 3000 Leuven, Belgium

6 present address: Max-Planck-Institute for Brain Research, Max-von-Laue-Strasse 4, D-60438 Frankfurt, Germany

7 present address: od green GmbH, Passauerstrasse 34, A-4780 Schärding am Inn, Austria

8 Institute of Cell Biology and Neuroscience, Goethe University, Max-von-Laue-Strasse 13, D-60439 Frankfurt, Germany

9 present address: Center for Computational and Theoretical Biology (CCTB), University of Würzburg, Campus Hubland Nord 32, D-97074 Würzburg, Germany

10 these authors contributed equally to this work

11 to whom correspondence should be addressed

## Supplementary Information

## Supplementary figures

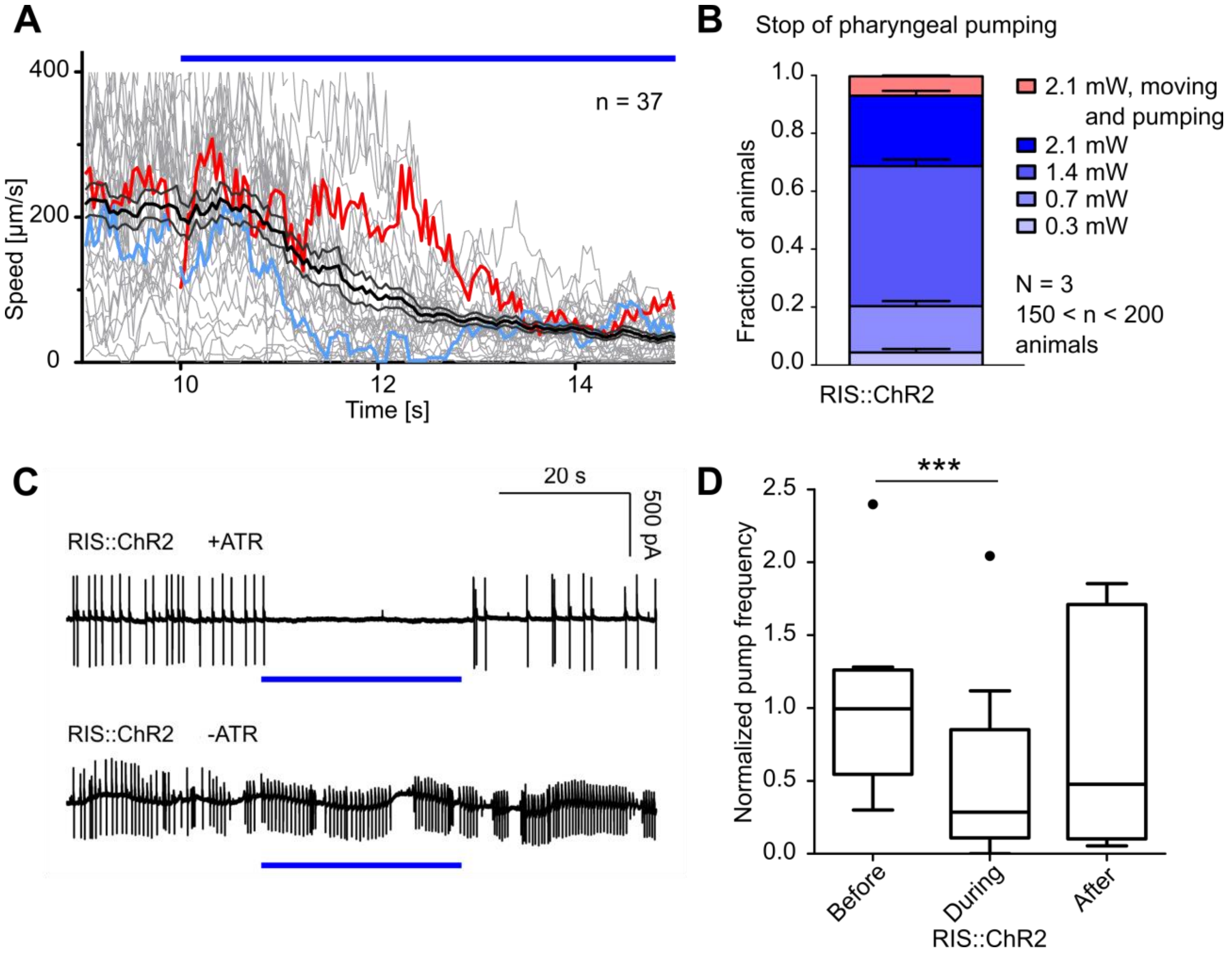


**Supplementary Figure 1: Stop behavior and pharyngeal inhibition, elicited by RIS photostimulation. A**) Locomotion stop delay is variable in WT animals. A fast (blue) and a slow (red) responder are highlighted. All other traces are shown in grey, the mean (thick line) ± SEM (thin lines) of all animals is shown in black. Illumination period is indicated by a blue bar. Zoomed in from data in **Fig. 1B**. **B**) Pharyngeal pumping inhibition upon RIS::ChR2 depolarization depends on illumination intensity. Pharynx pumping was visually inspected during RIS photostimulation, at different light intensities. The fraction of animals stopping pumping and locomotion at the indicated light intensities was registered for the indicated number of animals in three independent experiments. **C**) Electropharyngeograms obtained from cut-head preparation of RIS::ChR2 expressing animals, raised either with or without ATR. Blue bar indicates illumination (3 $mW/mm^2$, 470 nm). Spontaneous pumping was achieved by 5 min incubation with 2 µM serotonin prior to data acquisition. **D**) Normalized pump frequency of animals expressing RIS::ChR2 raised with ATR. Pump frequency aggregated per animal and 30 s bin as shown in (C). Box plot with Tukey whiskers. ***p≤0.001: Student's T-test with Bonferroni correction, n = 10.

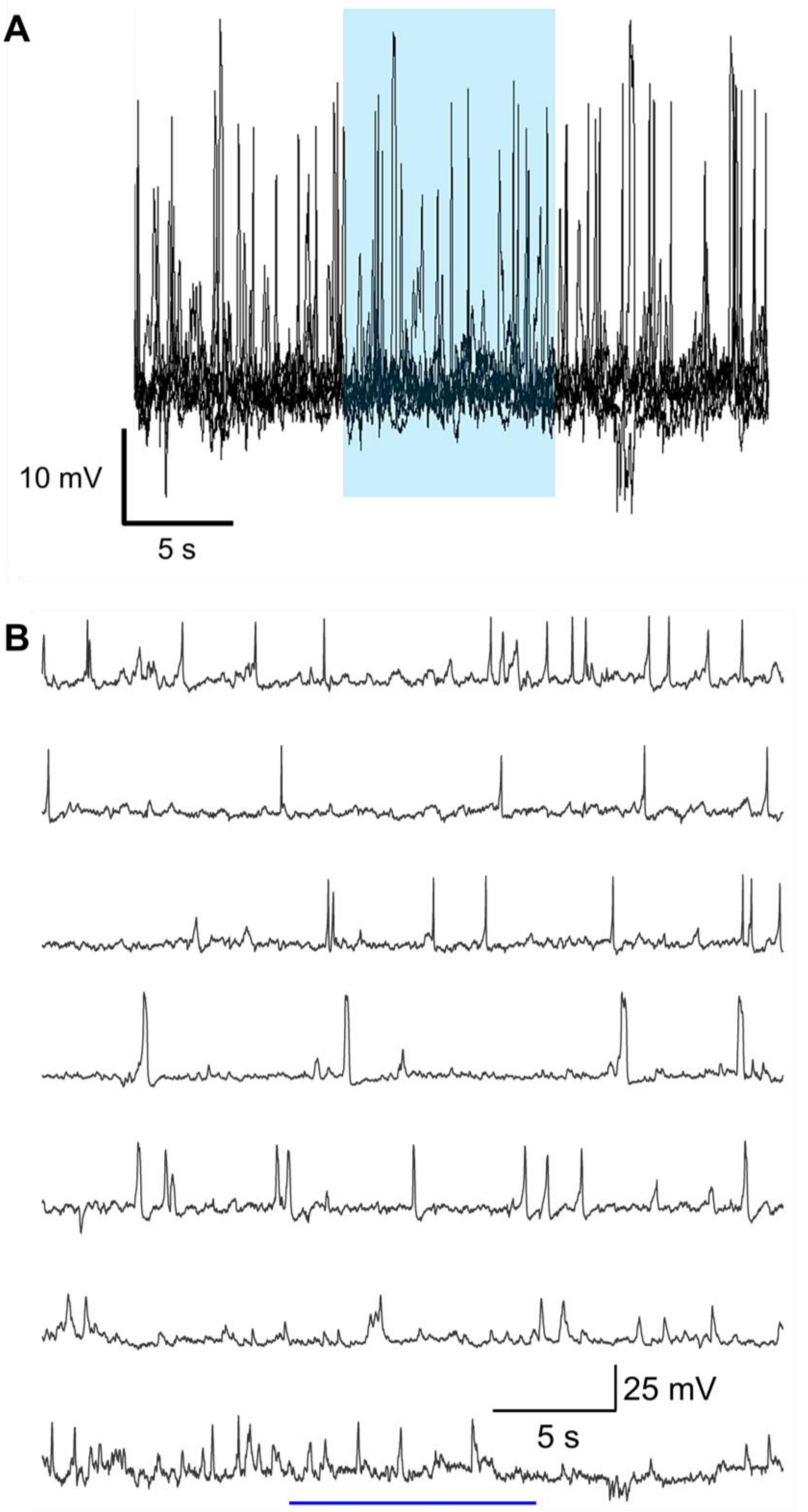


**Supplementary Figure 2: RIS photostimulation did not inhibit action potentials in BWMs: A)** Overlay of the seven single current clamp measurements recorded from BWM at the ventral side, anterior to the vulva, as shown in **B)**. RIS::ChR2 photostimulation denoted by blue bar and/or shading.

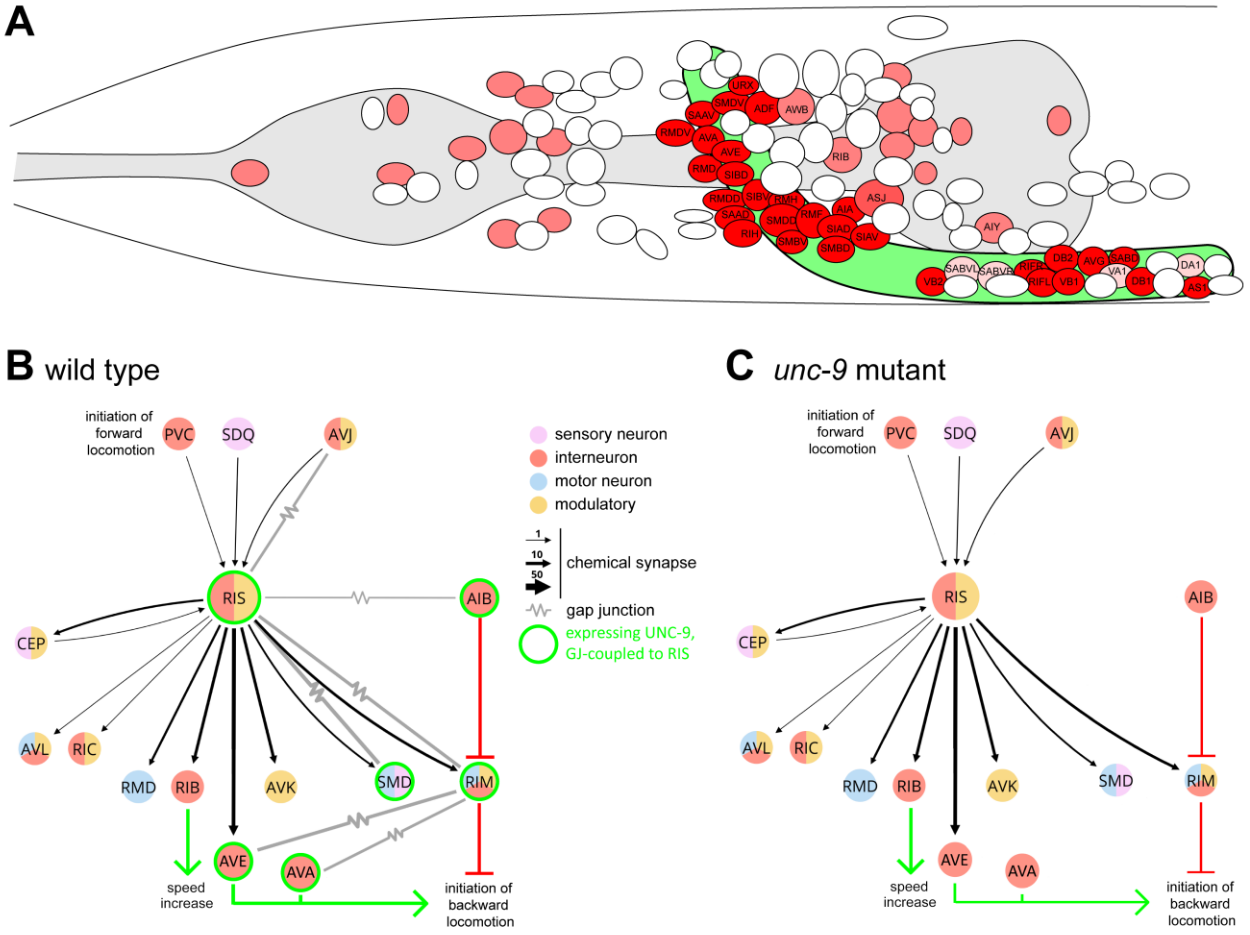


**Supplementary Figure 3: Neuron positions in the head and connectome of the RIS neuron. A**) Representation of the cholinergic cell bodies with the ROI scored in **Fig. 3E**, depicted as green shaded area. Pink cell bodies are cholinergic neurons outside of the imaged region, while their processes might influence the recorded signal as well. Based on Gendrel, M., Atlas, E.G. & Hobert, O. A cellular and regulatory map of the GABAergic nervous system of C. elegans. *eLife* **5**, e17686 (2016). **B)** Connectome of the RIS neuron, and selected further neurons connected indirectly to RIS. Arrows depict chemical synapses, thickness represents abundance of connections. Grey lines represent electrical synapses. DA neurons, also gap junction-coupled to RIS, are not shown. Neuron types are color coded, as indicated. Modified from nemanode.org, and based on White JG, Southgate E, Thomson JN, Brenner S. The Structure of the Nervous System of the Nematode Caenorhabditis elegans. Philos Trans R Soc Lond B Biol Sci 314, 1-340 (1986). The expression pattern of the UNC-9 innexins, based on Bhattacharya A, Aghayeva U, Berghoff EG, Hobert O. Plasticity of the Electrical Connectome of C. elegans. Cell 176, 1174-1189 e1116 (2019), is indicated by green circles around neurons (UNC-9 is only shown for neurons connected to RIS). **C)** As in (B) but omitting gap junctions mediated by UNC-9.

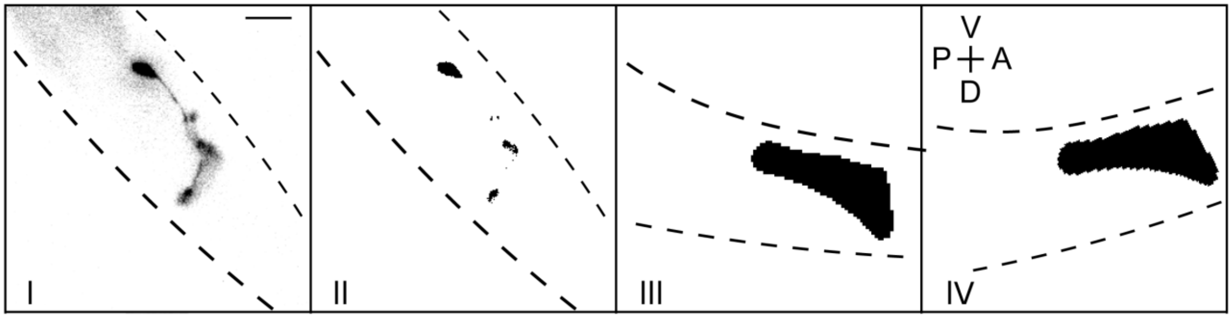


**Supplementary Figure 4. Initial steps in image processing for RIS axon $Ca^{2+}$ imaging: B**) Image analysis involved cropping (I), binarization (II), repositioning the soma (III), and determining the angular orientation of the axon (IV). D, dorsal; V, ventral; P, posterior; A, anterior. Scale bar: 20 µm.

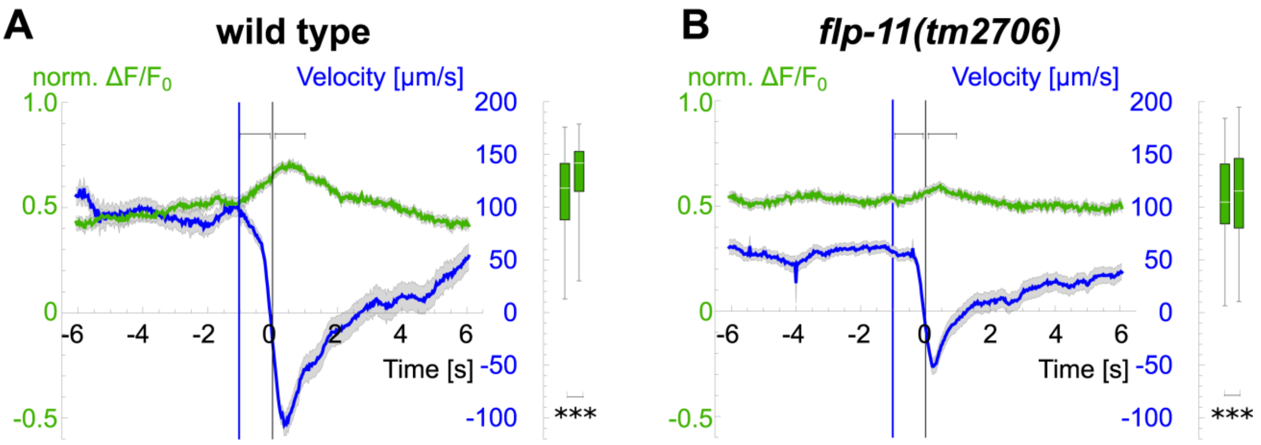


**Supplementary Figure 5. RIS $Ca^{2+}$ signals are aligned to the moment of reversal (zero velocity), compared in wild type and *flp-11(tm2706)* mutants: A)** Reversal-aligned normalized GCaMP fluorescence intensity (green) and animal velocity in µm/s (blue). A significant increase in $Ca^{2+}$ (n=75 reversals; p <0.001) was observed at the time before and after the moment of reversal, calculated for time periods indicated by brackets, flanking the point of zero velocity during reversal (blue lines). **B)** as in A), but in *flp-11(tm2706)* mutants. Significant increase in calcium (n=100 reversal events). Box plots with Tukey whiskers, ***p≤0.001.

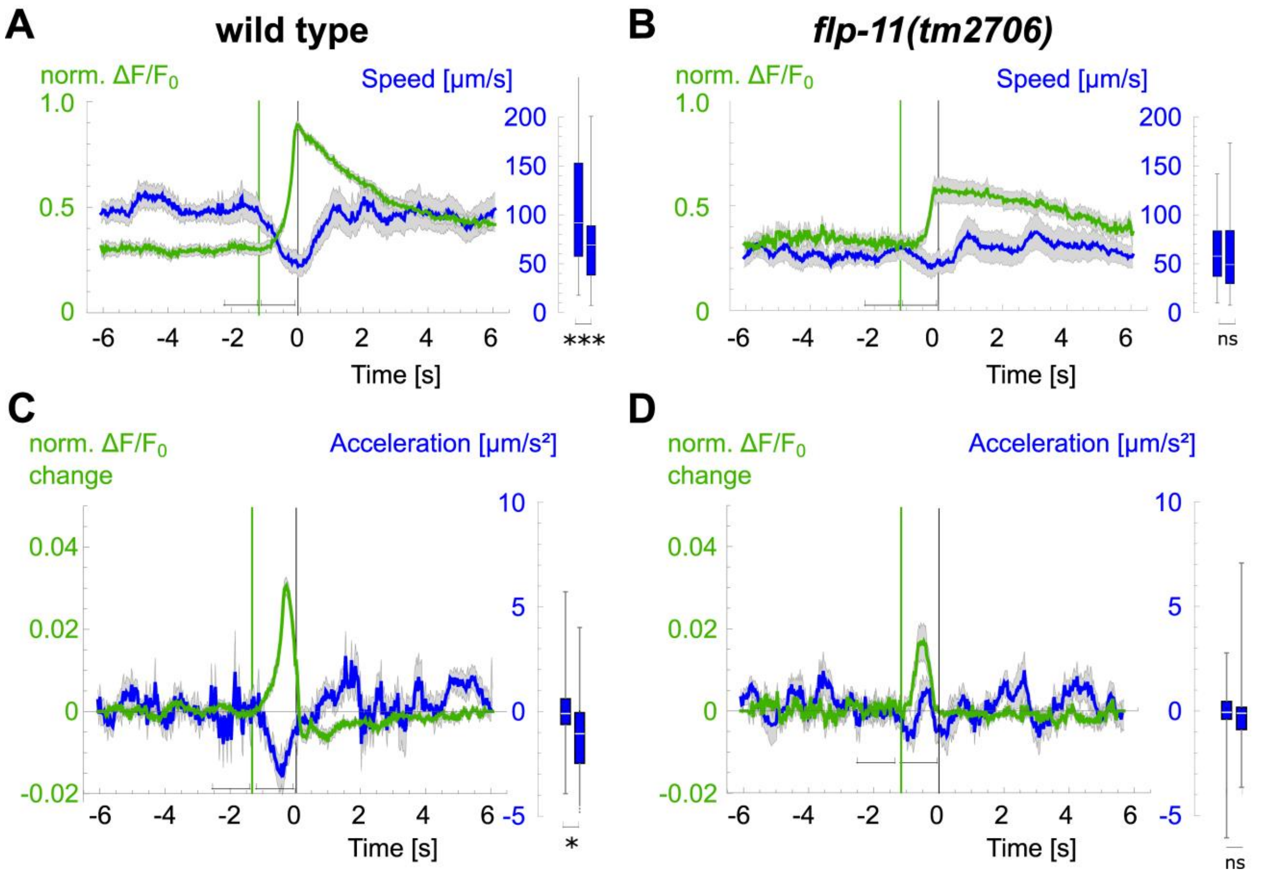


**Supplementary Figure 6. RIS $Ca^{2+}$ signals, or their time derivative, aligned to the peak, and concomitant animal speed or acceleration, compared in wild type and *flp-11(tm2706)* mutants: A, C)** $Ca^{2+}$ peak aligned normalized fluorescence (A), or (C) $Ca^{2+}$ change rate (dF/dt) (green), and the animal speed (A) or acceleration (C) in µm/s² (blue). n=45 $Ca^{2+}$ peaks were aligned. Significant reductions in speed or acceleration were observed at the times before and during the onset of $Ca^{2+}$ rise (indicated by green vertical lines). Differences were calculated for time periods indicated by brackets. Box plots with Tukey whiskers, ***p≤0.001; *p≤0.05. B, D) as in A, C), but for *flp-11(tm2706)* mutants (n=25 $Ca^{2+}$ peaks).

## Supplementary Movies

**Supplementary Movie 1. RIS photostimulation leads to a block of locomotion behavior:** Illumination of the anterior portion with a tracking and illumination system (Stirman et al., 2011) leads to a stop of the locomotion. Left half shows video stream, on the right, the illumination pattern sent to the projector is shown.

**Supplementary Movie 2. Photostimulation of GABA neurons leads to a transient inhibition of locomotion:** Stimulation of ChR2, expressed in GABA neurons (*punc-47* promoter), causes a transient stop followed by resumed, yet uncoordinated locomotion.

**Supplementary Movie 3. $Ca^{2+}$ fluctuations in the BWMs are suppressed by RIS photostimulation: A)** Animal expressing RCaMP1h in BWMs and ChR2 in RIS is immobilized and imaged for RCaMP fluorescence, shown in 'fire' lookup table. Video is accelerated 5 times. **B)** Difference video corresponding to A, showing fluorescence increase and decrease in red and blue, respectively.

**Supplementary Movie 4. $Ca^{2+}$ oscillations in cholinergic MNs are suppressed by RIS photostimulation:** Animal expressing RCaMP1h in cholinergic MNs and ChR2 in RIS is immobilized and imaged for RCaMP fluorescence in the head ganglia. Difference video, showing fluorescence increase and decrease in red and blue, respectively. Video is accelerated 20 times.

**Supplementary Movie 5. Depiction of workflow for video processing to deduce localized $Ca^{2+}$ dynamics in the RIS axon:** Upper row, from left to right: cropping, binarization, repositioning the soma, determining the angular orientation of the axon. Lower row: reorienting the raw image, masking unspecific gut fluorescence, fitting a parabola, measuring fluorescence intensity in perpendicular rectangular ROIs.

**Supplementary Movie 6. Example video of RIS activity during free locomotion, including slowing and reversal events**: Left half: RFP fluorescence (pharynx marker for tracking); right half: GCaMP6s image. Upper left corner: Reoriented and cropped image of RIS in the GCaMP6s channel.

**Supplementary Movie 7. RIS $Ca^{2+}$ activity in the nerve ring axon and in the branch during locomotion.** Left: original video of the head region of free moving *C. elegans* expressing GCaMP6s in RIS. Upper right: Extracted RIS neuron fluorescence, with indicated (right, below) morphology (CB: cell body, Br: branch, NR: nerve ring portion of the axon, with overlaid false-colored $Ca^{2+}$ fluorescence $\Delta F/F_0$ signal (blue – white hues: low – intermediate signal, yellow – red hues: intermediate – high signal. Scale and arrow on the right indicate locomotion speed and direction (green: forward, red: reverse).

**Supplementary Movie 8. Example video of RIS branch activity during free locomotion. A)** RIS GCaMP6s fluorescence signal false colored coded during a reversal. **B)** Analogous to A), but during a stop without subsequent reversal.

## Supplementary Information files:

Excel sheets with the original / processed data for each of the figure panels.
