# Supplementary material for: A GABAergic and peptidergic sleep neuron as a locomotion stop neuron with compartmentalized Ca2+ dynamics
Source: Nat Commun. 2019 Sep 10;10:4095. doi: 10.1038/s41467-019-12098-5 (PMC6736843; doi:10.1038/s41467-019-12098-5)
Supplement: Supplementary file 2 — Description of Additional Supplementary Files [file 41467_2019_12098_MOESM2_ESM.pdf]

Description of Additional Supplementary Files

File Name: Supplementary Movie 1
Description: RIS photostimulation leads to a block of locomotion behavior: Illumination of the anterior portion with a tracking and illumination system (Stirman et al., 2011) leads to a stop of the locomotion. Left half shows video stream, on the right, the illumination pattern sent to the projector is shown.

File Name: Supplementary Movie 2
Description: Photostimulation of GABA neurons leads to a transient inhibition of locomotion: Stimulation of ChR2, expressed in GABA neurons (punc-47 promoter), causes a transient stop followed by resumed, yet uncoordinated locomotion.

File Name: Supplementary Movie 3
Description: Ca2+ fluctuations in the BWMs are suppressed by RIS photostimulation: A) Animal expressing RCaMP1h in BWMs and ChR2 in RIS is immobilized and imaged for RCaMP fluorescence, shown in 'fire' lookup table. Video is accelerated 5 times. B) Difference video corresponding to A, showing fluorescence increase and decrease in red and blue, respectively.

File Name: Supplementary Movie 4
Description: Ca2+ oscillations in cholinergic MNs are suppressed by RIS photostimulation: Animal expressing RCaMP1h in cholinergic MNs and ChR2 in RIS is immobilized and imaged for RCaMP fluorescence in the head ganglia. Difference video, showing fluorescence increase and decrease in red and blue, respectively. Video is accelerated 20 times.

File Name: Supplementary Movie 5
Description: Depiction of workflow for video processing to deduce localized Ca2+ dynamics in the RIS axon: Upper row, from left to right: cropping, binarization, repositioning the soma, determining the angular orientation of the axon. Lower row: reorienting the raw image, masking unspecific gut fluorescence, fitting a parabola, measuring fluorescence intensity in perpendicular rectangular ROIs.

File Name: Supplementary Movie 6
Description: Example video of RIS activity during free locomotion, including slowing and reversal events: Left half: RFP fluorescence (pharynx marker for tracking); right half: GCaMP6s image. Upper left corner: Reoriented and cropped image of RIS in the GCaMP6s channel.

File Name: Supplementary Movie 7
Description: RIS Ca2+ activity in the nerve ring axon and in the branch during locomotion. Left: original video of the head region of free moving C. elegans expressing GCaMP6s in RIS. Upper right: Extracted RIS neuron fluorescence, with indicated (right, below) morphology (CB: cell body, Br: branch, NR: nerve ring portion of the axon, with overlaid false-colored Ca2+ fluorescence $\Delta F/F0$ signal (blue – white hues: low – intermediate signal, yellow – red hues: intermediate – high signal. Scale and arrow on the right indicate locomotion speed and direction (green: forward, red: reverse).

File Name: Supplementary Movie 8
Description: Example video of RIS branch activity during free locomotion. A) RIS GCaMP6s fluorescence signal false colored coded during a reversal. B) Analogous to A), but during a stop without subsequent reversal.
